# The efficiency of chronic disease care in sub-Saharan Africa

**DOI:** 10.1186/s12916-016-0675-6

**Published:** 2016-08-26

**Authors:** Pascal Geldsetzer, Katrina Ortblad, Till Bärnighausen

**Affiliations:** 1 Department of Global Health and Population, Harvard T.H. Chan School of Public Health, 665 Huntington Avenue, Building 1, Boston, MA 02115 USA; 2 Institute of Public Health, Heidelberg University, Im Neuenheimer Feld 324, Heidelberg, 69120 Germany; 3 Africa Health Research Institute, P.O. Box 198, Mtubatuba, 3935 South Africa

**Keywords:** Efficiency, Differentiated care, Integrated care, Chronic diseases, Antiretroviral therapy, HIV, Non-communicable diseases

## Abstract

The number of people needing chronic disease care is projected to increase in sub-Saharan Africa as a result of expanding human immunodeficiency virus (HIV) treatment coverage, rising life expectancies, and lifestyle changes. Using nationally representative data of healthcare facilities, Di Giorgio et al. found that many HIV clinics in Kenya, Uganda, and Zambia appear to have considerable untapped capacity to provide care for additional patients. These findings highlight the potential for increasing the efficiency of clinical processes for chronic disease care at the facility level. Important questions for future research are how estimates of comparative technical efficiency across facilities change, when they are adjusted for quality of care and the composition of patients by care complexity. Looking ahead, substantial research investment will be needed to ensure that we do not forgo the opportunity to learn how efficiency changes, as chronic care is becoming increasingly differentiated by patient type and integrated across diseases and health systems functions.

Please see related article: http://bmcmedicine.biomedcentral.com/articles/10.1186/s12916-016-0653-z

## Evolving health systems for chronic care in sub-Saharan Africa

The disease burden of chronic non-communicable diseases (NCD) is increasing in sub-Saharan Africa (SSA), while the burden attributable to acute infectious diseases is on the decline [1]. In the midst of this epidemiological transition, increased access to effective antiretroviral therapy (ART) is transforming human immunodeficiency virus (HIV) infection from a disease that cuts life expectancy by decades to a chronic affliction that does not substantially reduce life expectancy [2, 3]. As a result, the number of people needing chronic disease care continues to grow in the region [1, 4], calling into question the capacity of the generally weak health systems in SSA to effectively treat and support all patients needing chronic care [5, 6]. As ART continues to be scaled up with expanded eligibility [7], this strain on health systems in SSA will likely continue to increase over the coming decades [7].

The health system challenges posed by the continuously increasing number of patients with HIV have spurred efforts to assess the efficiency of current models of HIV care as well as to develop new, more efficient models of care. HIV and NCD share many health system challenges in terms of the successful implementation of primary prevention, screening, and early linkage to care; monitoring of therapeutic success; and the need for long-term medication adherence. Thus, research on the efficiency of health systems in delivering HIV care will also provide crucial lessons for health systems to help them successfully address the expected rise in the burden of NCD. This commentary discusses the recent article by Di Giorgio et al. in *BMC Medicine* [8], and future areas of research that emerge from this work.

## Untapped capacity in health systems in sub-Saharan Africa

The study by Di Giorgio et al. suggests that the health systems in Kenya, Uganda, and Zambia have considerable capacity to deliver HIV care to more patients without adding labor and capital inputs [8]. In fact, the authors estimate that the current systems could deliver care to an additional 459,000 patients with HIV– a 40 % increase in the current HIV patient volume. These findings imply that many healthcare facilities in the study countries have untapped capacity to deal with not only higher volumes of HIV patients, but also increased demand for other types of chronic disease care.

Thus far, policy and research in the field of healthcare delivery in SSA has largely been concerned either with inducing demand for healthcare, or, more recently, improving the quality of care on the supply side. The study by Di Giorgio et al. is an important contribution to this field because it points to a research and policy area that has received little attention thus far: supply-side interventions at the level of the healthcare facility to increase the efficiency of care.

We see two important methods improvements to increase the robustness of this research: controlling for quality of care and controlling for patient composition when measuring efficiency of care. We also see the emergence of a new focus in health systems research: experiments and quasi-experiments to establish the causal impact of changing models of care on efficiency.

## Efficiency and quality of care

As healthcare facilities increase the quantity of care with a fixed level of inputs, quality of care may deteriorate. For instance, healthcare workers may have less time available to spend per patient, resulting in lower diagnostic accuracy and reduced patient satisfaction. One important area of future research will thus be to carefully assess the degree to which the volume of chronic care patients can be increased without adversely affecting quality of care and patient outcomes. Owing to data constraints, Di Giorgio et al. were only able to include structural indicators of care quality provided by healthcare facilities (e.g., the availability of certain medications). Future research in this area should control for outcome quality of care in the comparison of efficiency of care across facilities, including objective outcome quality (e.g., viral load suppression for HIV care, hemoglobin A1c for diabetes care) and subjective outcome quality (e.g., overall patient satisfaction with care). In addition, time-and-motion studies can provide insights into process aspects of quality of care by enumerating the health worker activities during the patient encounter and the time spent per activity [9].

## Patient composition

Patients in advanced disease stages or those with comorbidities are likely to demand more human resource inputs (in terms of both health worker time and skill level) and other inputs (e.g., blood tests) than healthier patients. The composition of the patient population – that is, the distribution of patients across ‘complex’ and ‘simple’ patient types – is therefore likely to have profound effects on the quantity of patients any given number of health workers can provide with high-quality care. Because patient composition is likely to vary between healthcare facilities, and at facilities over time, it will be important for future research on chronic care efficiency in SSA to adjust efficiency estimates for patient composition. Indicators of patient complexity will need to be carefully constructed to avoid systematic misclassification of complexity by quality of care. For instance, CD4-cell count is both an indicator of quality of care for patients with HIV, as well as a measure of HIV disease severity and thus patient complexity. While adjustments for patient composition will require additional data collection and research investments, we would argue that these adjustments are essential to accurately determine which healthcare facilities operate at the production possibility frontier and which are less efficient.

## New models of care

Recently, there has been a proliferation of new HIV care models, which provide varying levels of intensity and types of care to different categories of patients. Such models may deliver increased levels of facility-based care to some patients, such as adherence support for those who first present to HIV programs while feeling well [7]. However, for those who are clinically stable on ART, differentiated HIV care models usually aim to reduce patient volumes at healthcare facilities. Examples of such models are less frequent clinic appointments, the delivery of antiretroviral drugs (ARV) to patients’ homes, peer educator-led ARV refill groups, and community ARV distribution points [7, 10]. The finding by Di Giorgio et al. of untapped capacity in the current standard facility-based models of HIV care should be viewed in the context of these emerging models, which often aim to increase the efficiency of HIV care by decongesting healthcare facilities. Many of the new models are still in the development stage and have yet to be evaluated rigorously and at scale [10]. Measuring quality of care will be particularly critical in these evaluations because, in the new models of HIV care, highly qualified or specialized (and thus expensive) healthcare resources – such as nurses – are often replaced by less qualified or specialized (and thus less expensive) resources – such as community health workers. If the quality of HIV care using the new models of HIV care is at least equal to the quality of care delivered using the current models, the new models will be more efficient and should thus become the preferred approach.

Another change in HIV care in SSA is the joint delivery of HIV and NCD treatment in integrated models of chronic care [11]. Such integration is likely to affect efficiency of care in complex ways whose net effects are hard to predict and thus need to be empirically determined. From the perspective of the patient, integrated models of chronic care are likely efficiency-enhancing because they create a ‘one-stop shop’ for multiple chronic care needs (such as for HIV, diabetes, hypertension, and depression [12, 13]), lowering the number of healthcare visits each patient has to make and thus reducing average out-of-pocket expenditures and lost time from work to seek care [14]. The potential for such patient-centered efficiency gains is likely to increase over the coming decades as continuously rising ART coverage will increase the co-prevalence of HIV and NCD. This increase will be driven by a new epidemiological transition in SSA through several important pathways [2] (Geldsetzer et al. in press): ART allows patients with HIV to survive into the older ages when NCD become very common [3]; the aging process in people surviving on ART is accelerated by chronic HIV inflammation that is not eliminated by ART [15–17]; and several ARVs have side effects that cause NCD [18].

From the perspective of the health system, the impact of integrating HIV care with other health system functions is less clear. On the one hand, integration of care could reduce clinicians’ efficiency, because it decreases specialization and increases task switching costs, as suggested by classic division labor theory [19–21]. On the other hand, integration could also improve the efficiency of the health system through the joint utilization of fixed factors of production (e.g., clinics) and sharing of health system functions (e.g., monitoring and evaluation systems) [22]. In addition, it has been argued that integration will improve the quality of care through a shift from disease- to more person-centered healthcare [6], resulting in more trusting relationships between patient and health worker, and health workers who are more familiar with a patient’s multiple healthcare needs.

## Conclusions

The increasing demand for chronic diseases is a formidable challenge to health systems in SSA. Finding ways to improve efficiency of chronic disease care while maintaining or even increasing quality of care will be crucial. The empirical literature on this topic is astoundingly sparse; yet, the answers to many of the key questions on the efficiency of novel models for chronic care are far from obvious. Thus, we agree with Davies and colleagues who have recently concluded in a commentary on the future of chronic disease care in SSA that “[t]he lynchpin of a successful effort to grow health systems that can deal with communicable diseases and NCDs equally effectively is research” [23].
